# Supplementary material for: Small molecule screen in embryonic zebrafish using modular variations to target segmentation
Source: Nat Commun. 2017 Dec 1;8:1901. doi: 10.1038/s41467-017-01469-5 (PMC5711842; doi:10.1038/s41467-017-01469-5)
Supplement: Supplementary file 1 — Supplementary information [file 41467_2017_1469_MOESM1_ESM.pdf]

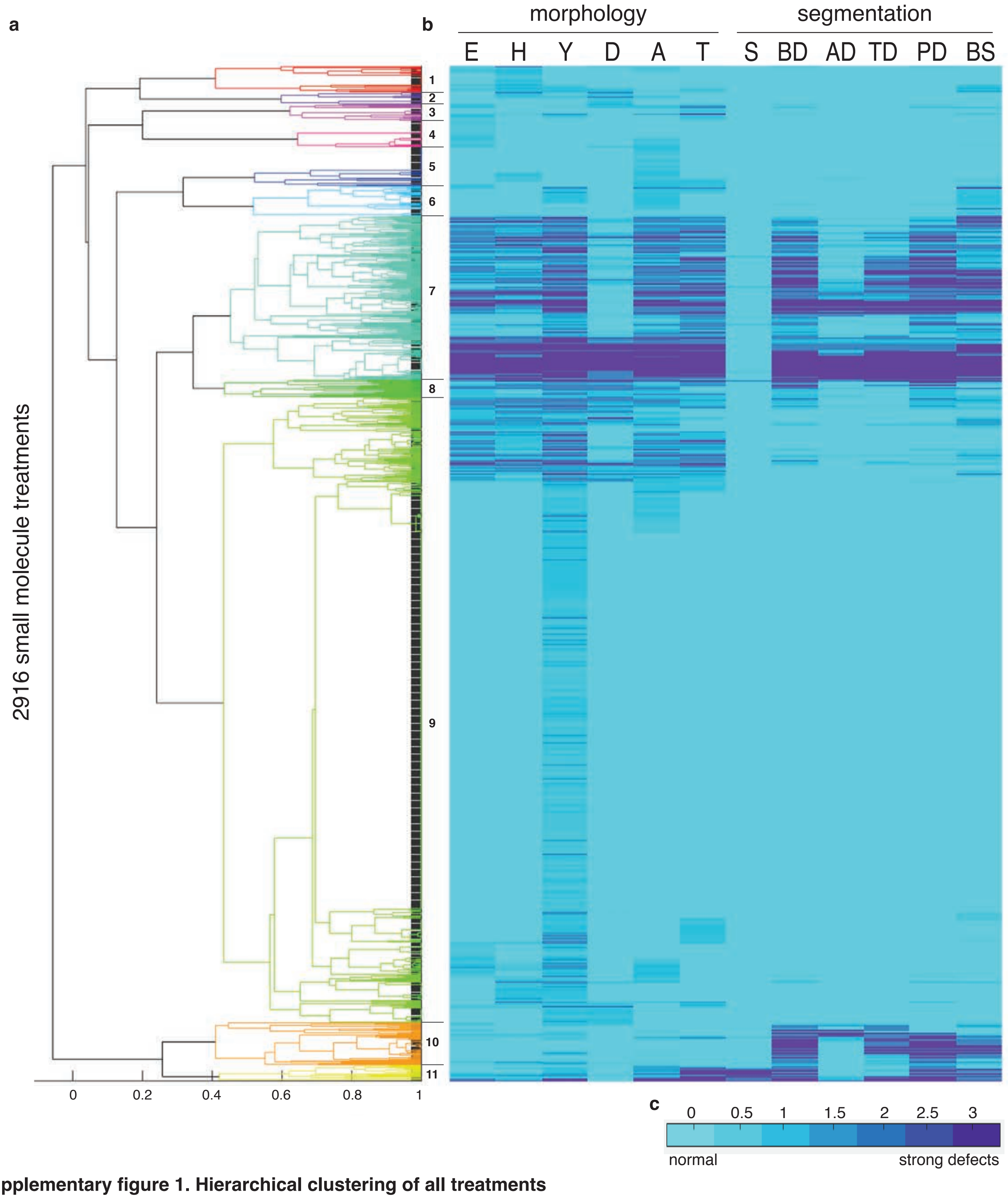

Supplementary figure 1. Hierarchical clustering of all treatments

(**a**) A dendrogram of phenotypic vectors clustered by their similarity. All 2916 small molecule treatments were clustered on the basis of their morphological and segmentation parameters. We found 11 different clusters of phenotypes (correlation cutoff of 0.6), indicated by coloured branches of the dendrogram and corresponding numbers. The three clusters of interest discussed in the main text are numbered 7, 10 and 11. (**b**) Criteria for phenotypic scoring based on morphological (embryonic development [E], head region [H], yolk [Y], dorsal-ventral development [D], axis elongation [A], tail shape [T]) and segmentation parameters (secondary tail appendage [S], myotome boundary defects [BD], anterior myotome defects [AD], trunk myotome defects [TD], posterior myotome defects [PD], myotome boundary shape [BS]). Each are rated from 0 to 3 (normal to strong difference) compared to untreated controls. Numerical parameter score converted into a colour scale bar (**c**). All parameters together generate a phenotypic vector for each treatment. Horizontal position of each treatment in the dendogram (**a**) corresponds to a phenotypic vector at the same horizontal level in (**b**).

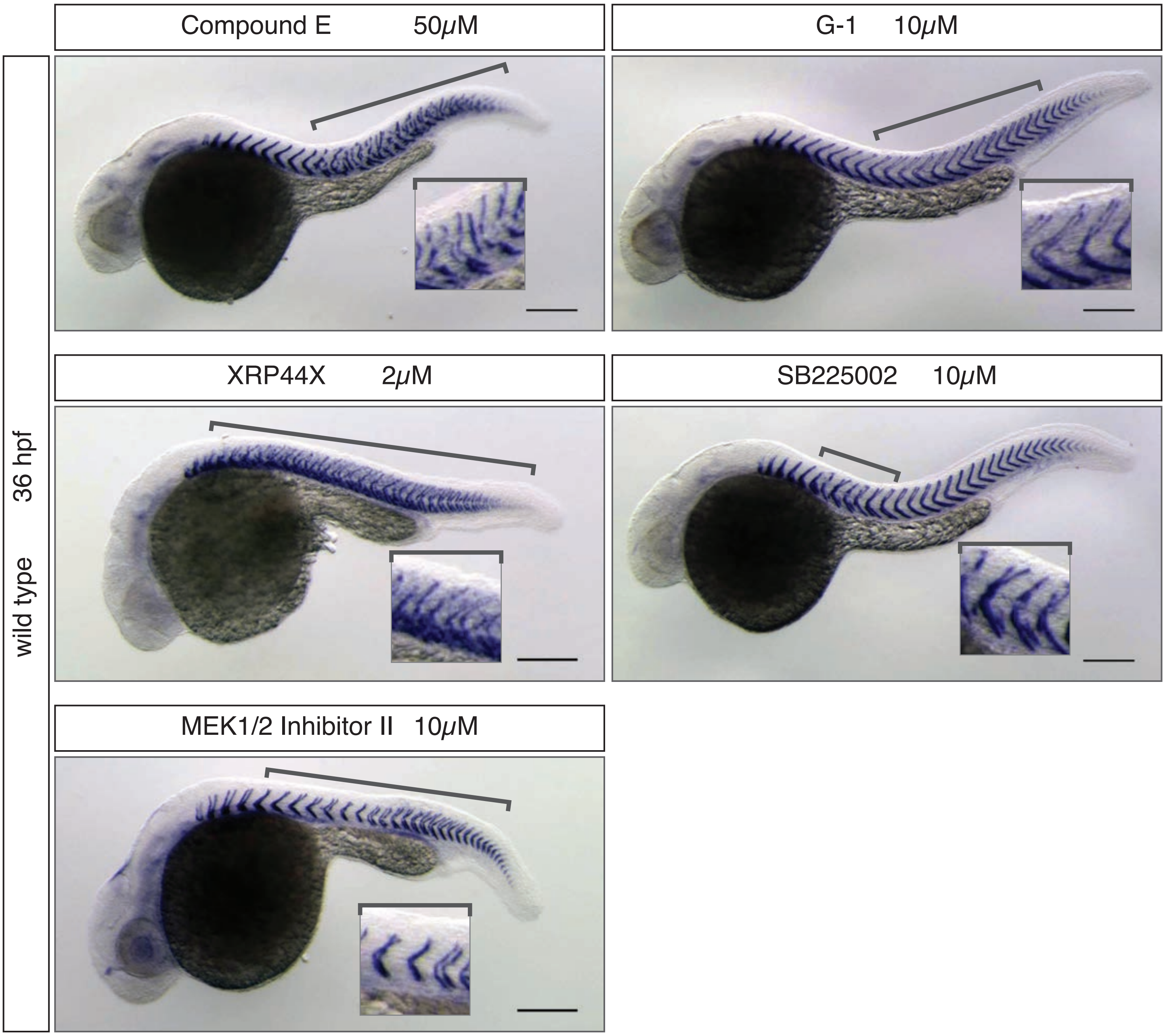

Supplementary figure 2. 1st class segmentation phenotypes
Identified hit phenotypes with direct segmentation defects according the classification used in Table 1, visible at 36 hpf after *xirp2a in situ* hybridization. All treatments are as labelled in the panel above the image. Anterior to the right, posterior to the left. Insets show zoom-in of the segment pattern and defects. Brackets indicate the area of the axis where segment boundary defects occurred.

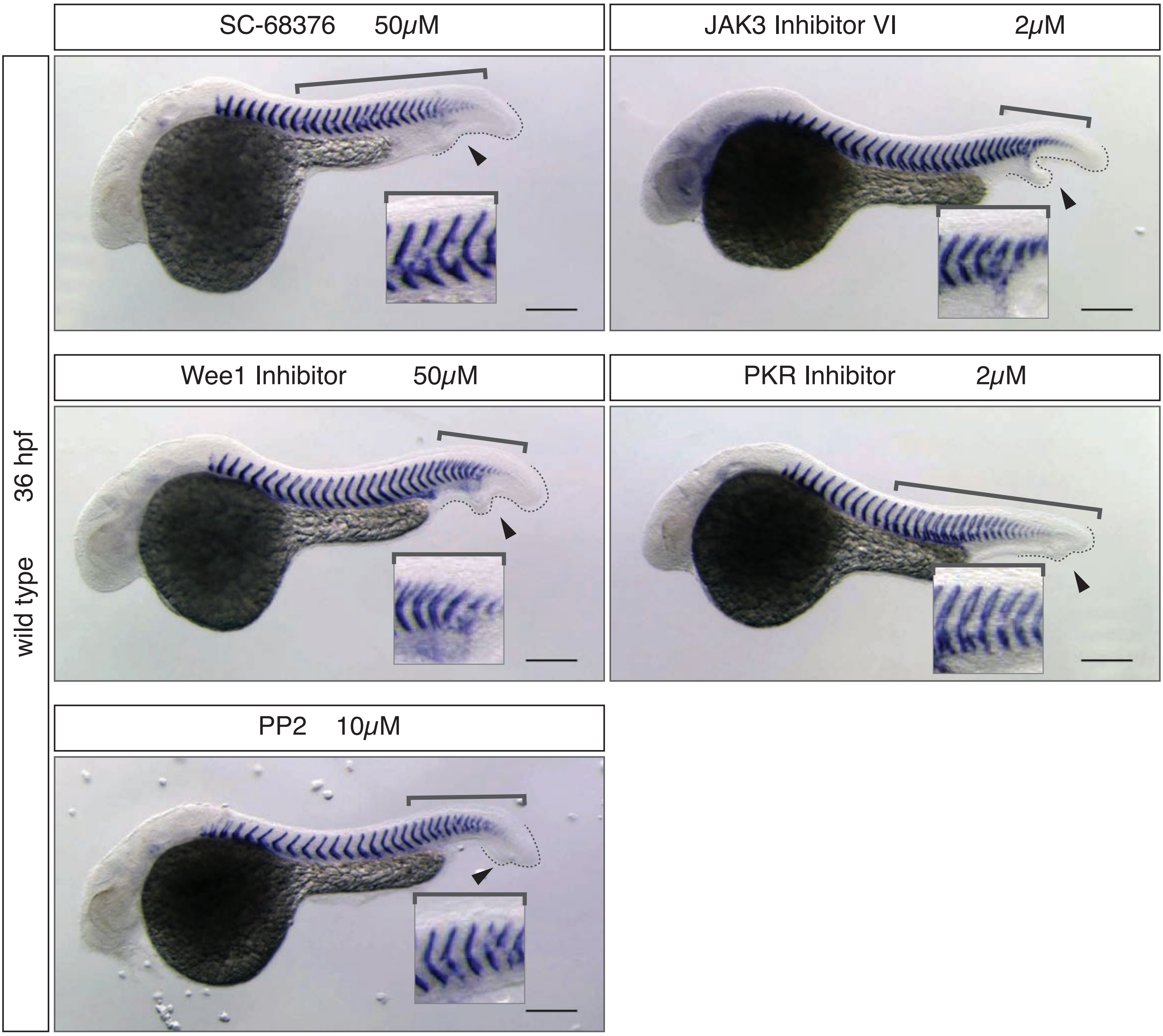

Supplementary figure 3. Secondary tail appendage phenotypes

Identified hit phenotypes with direct segmentation defects according the classification used in Table 1, visible at 36 hpf after *xirp2a in situ* hybridization. All treatments are as labelled in the panel above the image. Anterior to the right, posterior to the left. Insets show zoom-in of the segment pattern and defects. Brackets indicate the area of the axis where segment boundary defects occurred. Dotted line outlines observed secondary tail appendage.

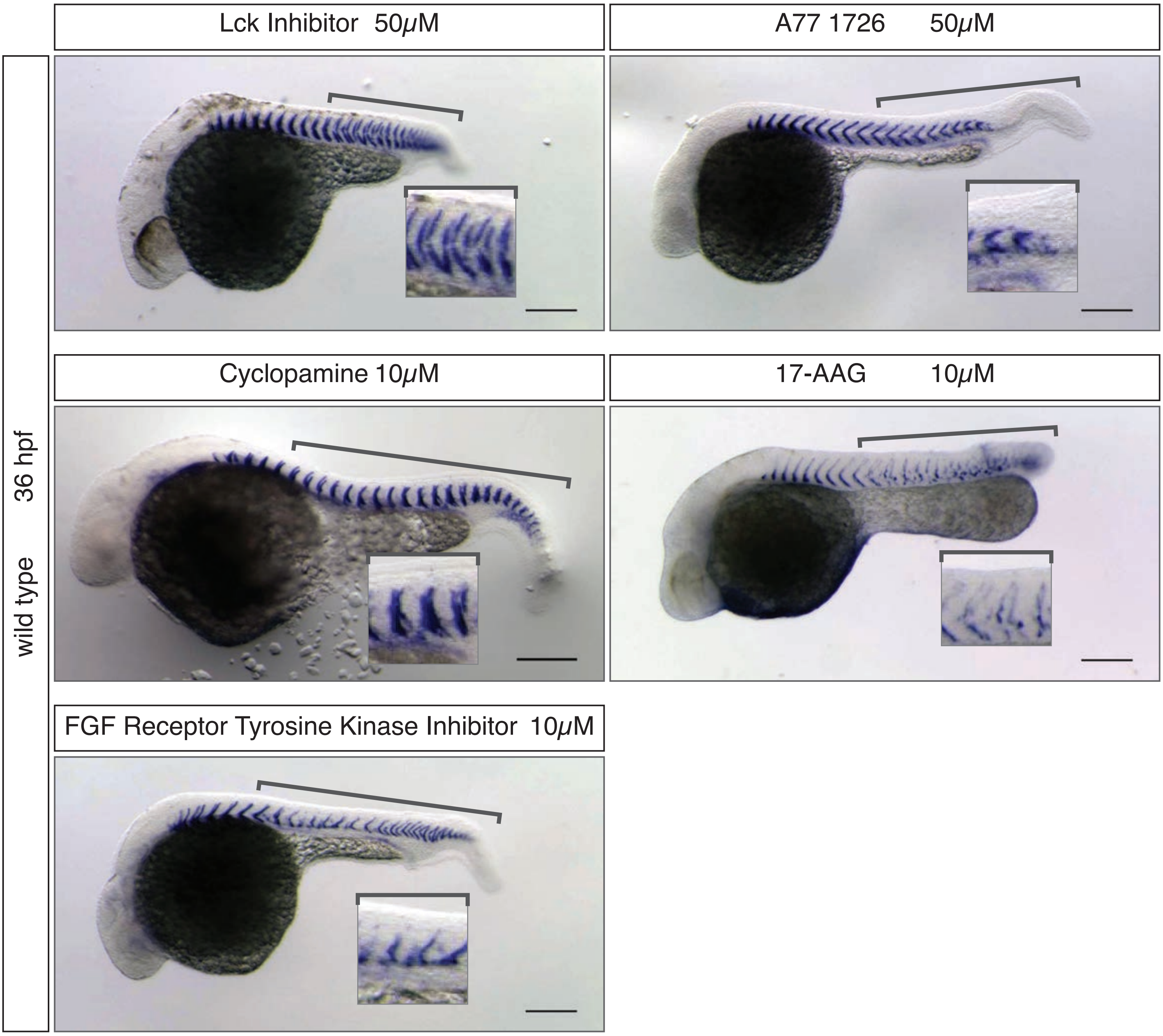

Supplementary figure 4. Phenotypes with lower segmentation-specificity

Identified hit phenotypes with direct segmentation defects according the classification used in Table 1, visible at 36 hpf after *xirp2a in situ* hybridization. All treatments are as labelled in the panel above the image. Anterior to the right, posterior to the left. Insets show zoom-in of the segment pattern and defects. Brackets indicate the area of the axis where segment boundary defects occurred.

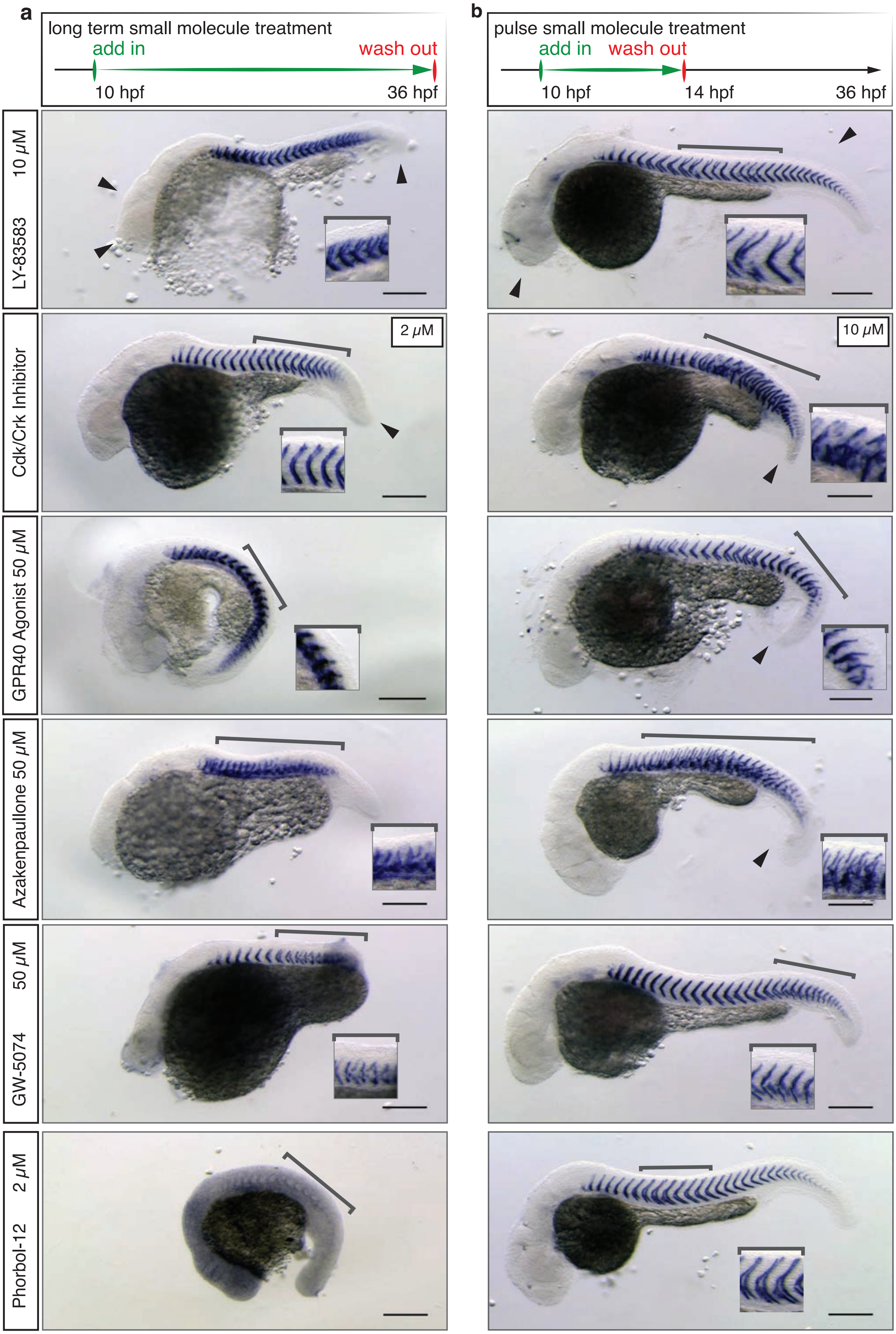

Supplementary figure 5. Pulse treatments with higher segmentation-specificity

Hits previously hidden in the general morphology cluster that were identified after 4h pulse treatment with subsequent wash-out. Small molecules and concentration used are labelled in the left panel. Anterior to the right, posterior to the left of each image. (**a**) Embryonic phenotypes as observed after long-term treatment during screen. (**b**) Embryonic phenotypes showing higher segmentation-specificity after shorter pulse treatment. In the case of the Cdk/Crk inhibitor, the 10 μM long-term treatment was lethal, so the 2 μM long-term treatment is shown instead. Insets show zoom-in of the segment pattern and defects. Brackets indicate the area of the axis where segment boundary defects occurred. Scale bar is 200 μm.

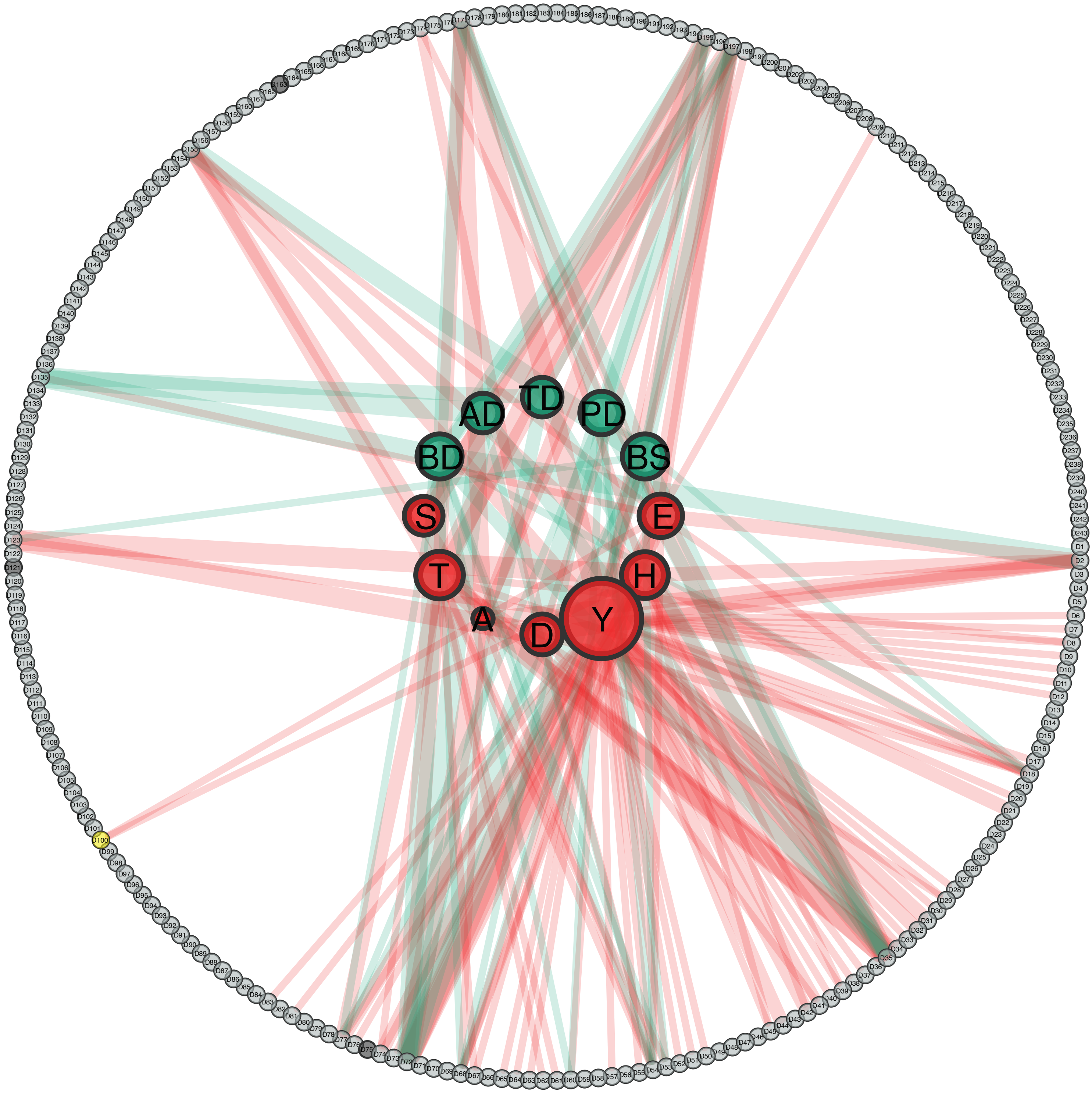

Supplementary figure 6. Network visualisation of phenotypic parameters wild type A, 2 μM

Phenotypic scoring per small molecule concentration illustrated as interaction networks. Inner circles represent phenotypic parameters, with morphology in green and segmentation in red. The size of nodes represents the frequency of scoring >0 for the individual parameter. Interactions to the outer circle represent the scoring of a phenotypic parameter to the associated small molecule treatment (grey circles). A legend of corresponding small molecule names can be found in Supplementary Data 1. Thickness of the interaction lines reflects the magnitude of the score. Dark grey circles represent lethal conditions. The networks are presented with vector graphics, so the details of numbers and interactions can be seen by zooming in.

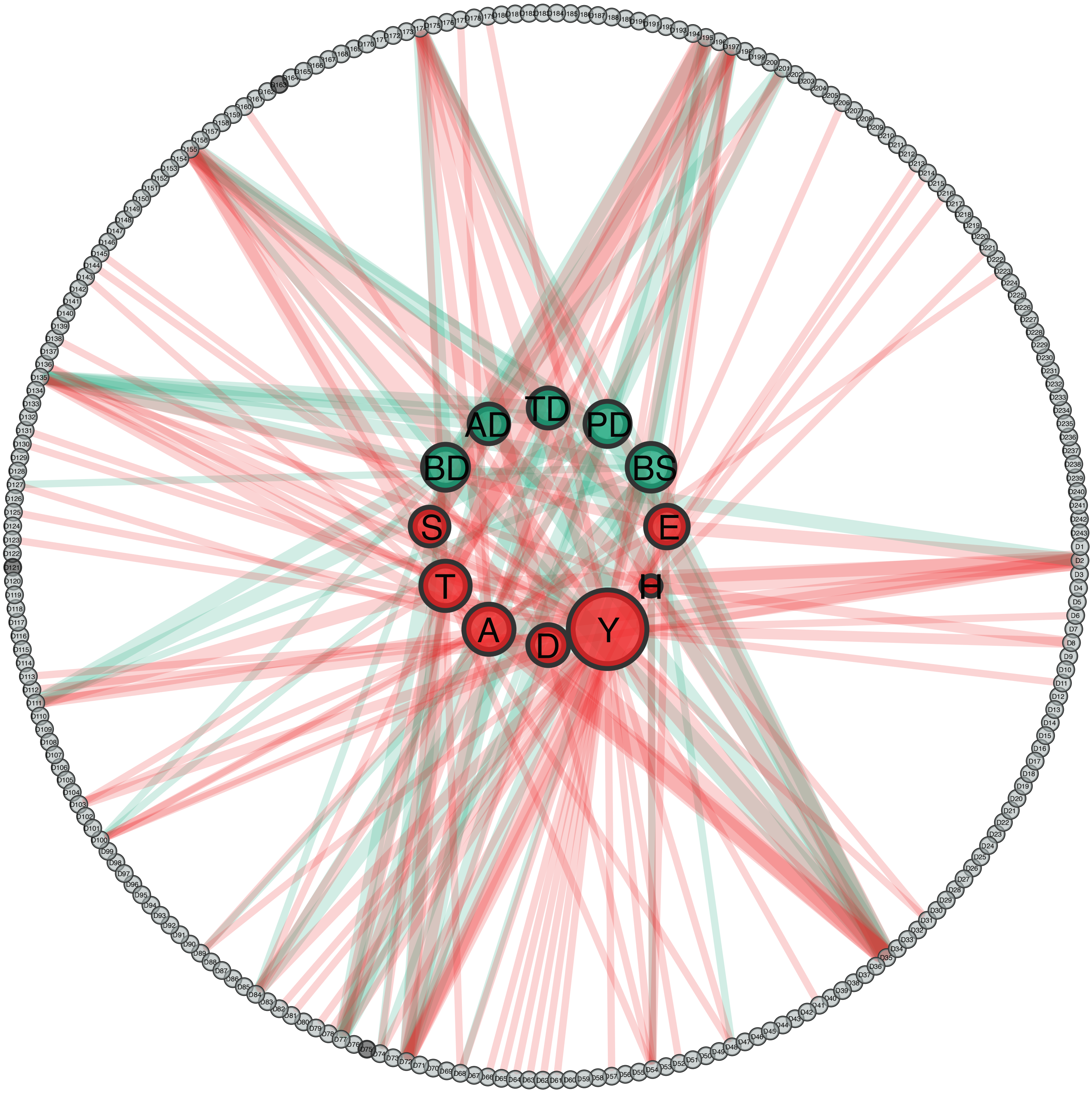

Supplementary figure 7. Network visualisation of phenotypic parameters wild type B, 2 μM

Phenotypic scoring per small molecule concentration illustrated as interaction networks. Inner circles represent phenotypic parameters, with morphology in green and segmentation in red. The size of nodes represents the frequency of scoring >0 for the individual parameter. Interactions to the outer circle represent the scoring of a phenotypic parameter to the associated small molecule treatment (grey circles). A legend of corresponding small molecule names can be found in Supplementary Data 1. Thickness of the interaction lines reflects the magnitude of the score. Dark grey circles represent lethal conditions. The networks are presented with vector graphics, so the details of numbers and interactions can be seen by zooming in.

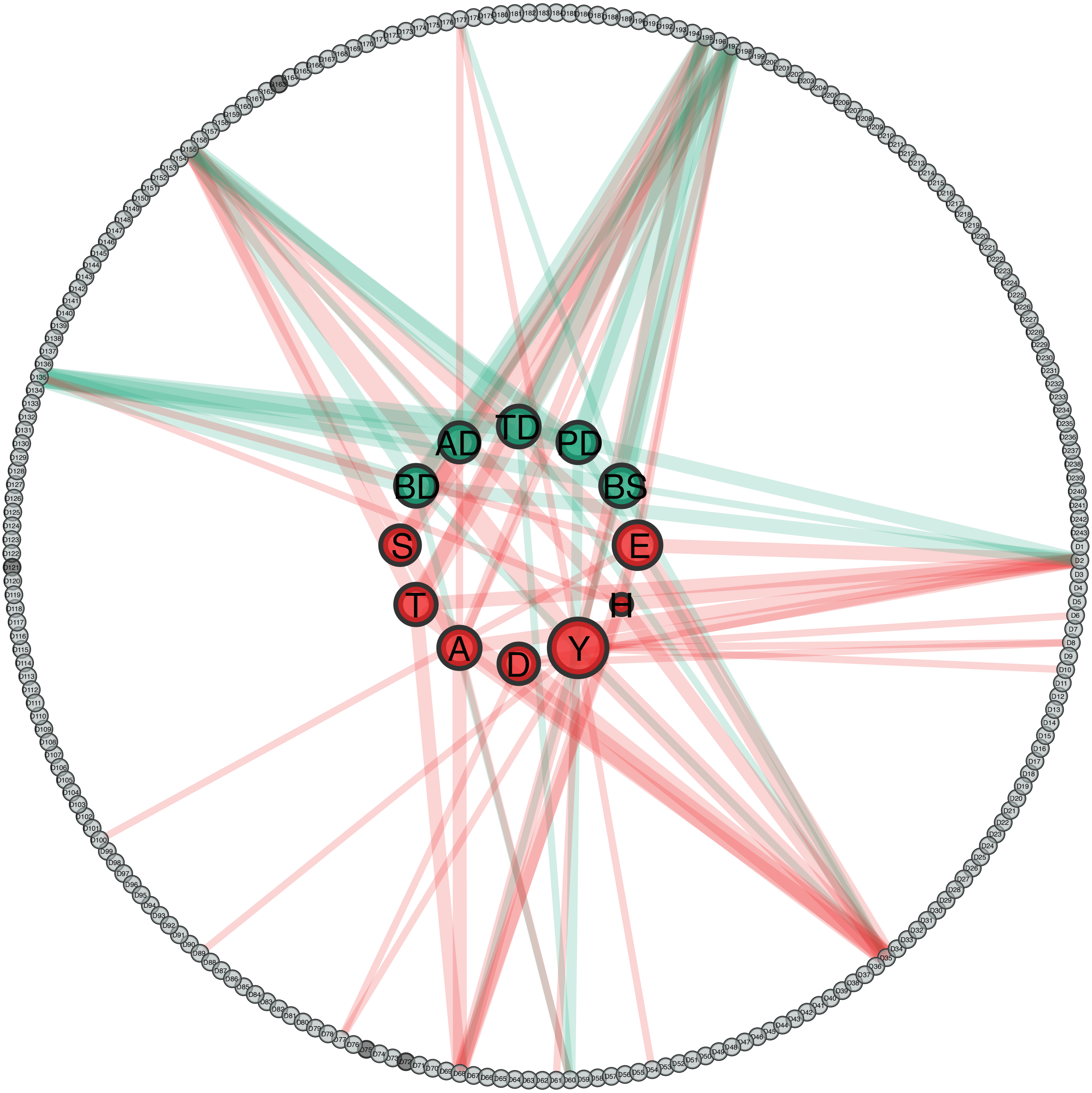

Supplementary figure 8. Network visualisation of phenotypic parameters *her1* mutant, 2 μM
Phenotypic scoring per small molecule concentration illustrated as interaction networks. Inner circles represent phenotypic parameters, with morphology in green and segmentation in red. The size of nodes represents the frequency of scoring >0 for the individual parameter. Interactions to the outer circle represent the scoring of a phenotypic parameter to the associated small molecule treatment (grey circles). A legend of corresponding small molecule names can be found in Supplementary Data 1. Thickness of the interaction lines reflects the magnitude of the score. Dark grey circles represent lethal conditions. The networks are presented with vector graphics, so the details of numbers and interactions can be seen by zooming in.

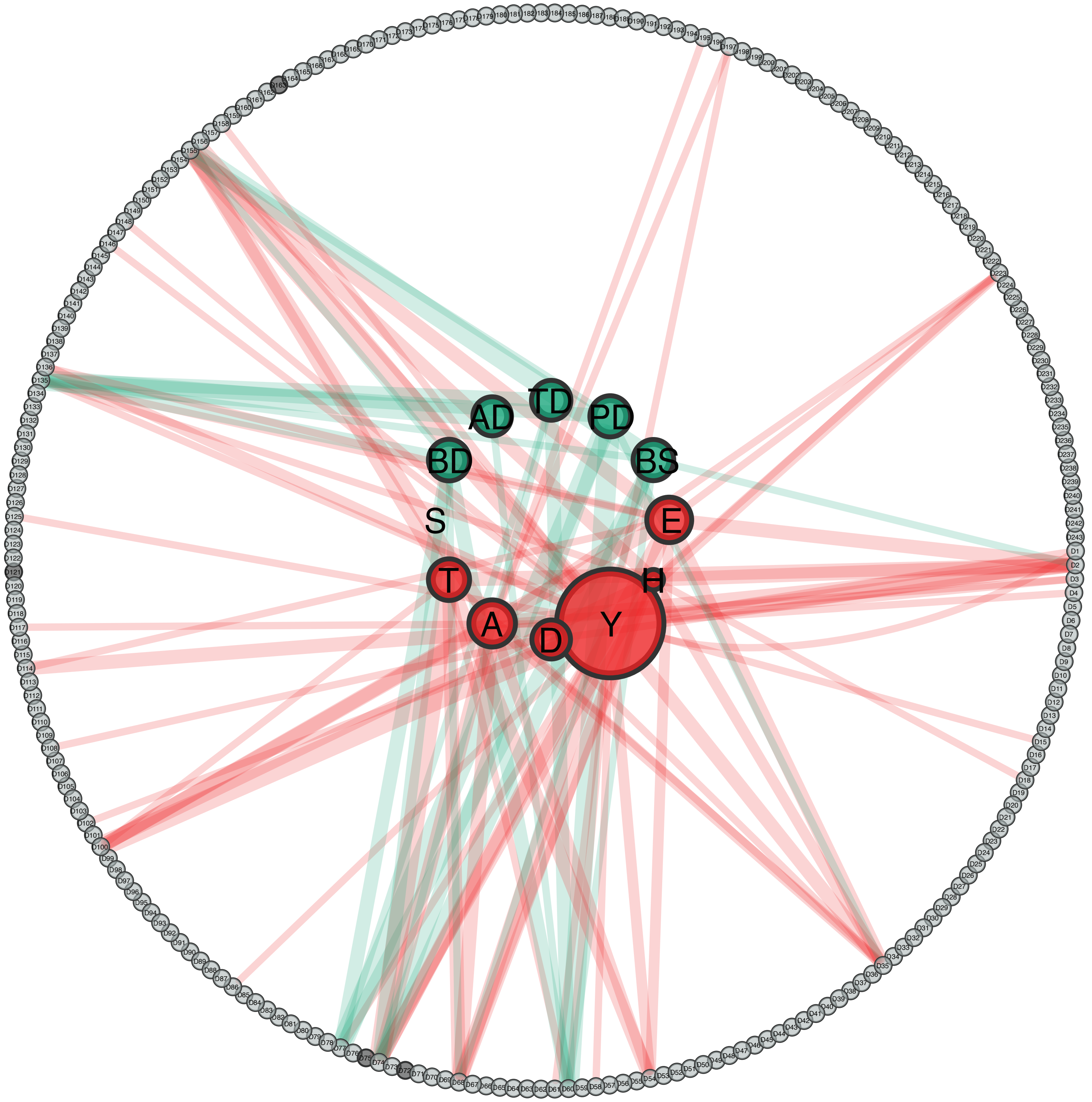

Supplementary figure 9. Network visualisation of phenotypic parameters *hes6* mutant, 2μM
Phenotypic scoring per small molecule concentration illustrated as interaction networks. Inner circles represent phenotypic parameters, with morphology in green and segmentation in red. The size of nodes represents the frequency of scoring >0 for the individual parameter. Interactions to the outer circle represent the scoring of a phenotypic parameter to the associated small molecule treatment (grey circles). A legend of corresponding small molecule names can be found in Supplementary Data 1. Thickness of the interaction lines reflects the magnitude of the score. Dark grey circles represent lethal conditions. The networks are presented with vector graphics, so the details of numbers and interactions can be seen by zooming in.

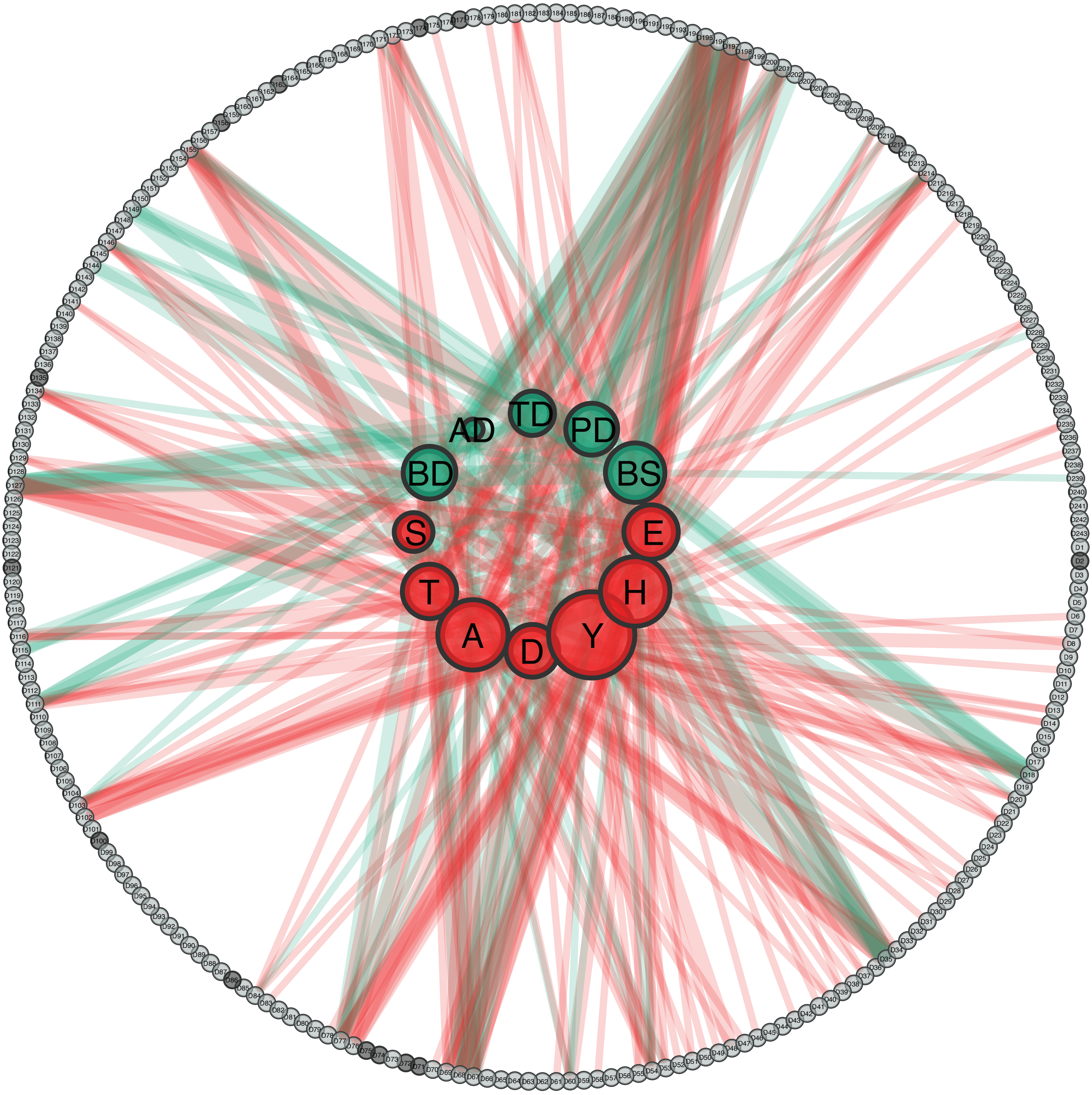

Supplementary figure 10. Network visualisation of phenotypic parameters wild type A, 10 μM

Phenotypic scoring per small molecule concentration illustrated as interaction networks. Inner circles represent phenotypic parameters, with morphology in green and segmentation in red. The size of nodes represents the frequency of scoring >0 for the individual parameter. Interactions to the outer circle represent the scoring of a phenotypic parameter to the associated small molecule treatment (grey circles). A legend of corresponding small molecule names can be found in Supplementary Data 1. Thickness of the interaction lines reflects the magnitude of the score. Dark grey circles represent lethal conditions. The networks are presented with vector graphics, so the details of numbers and interactions can be seen by zooming in.

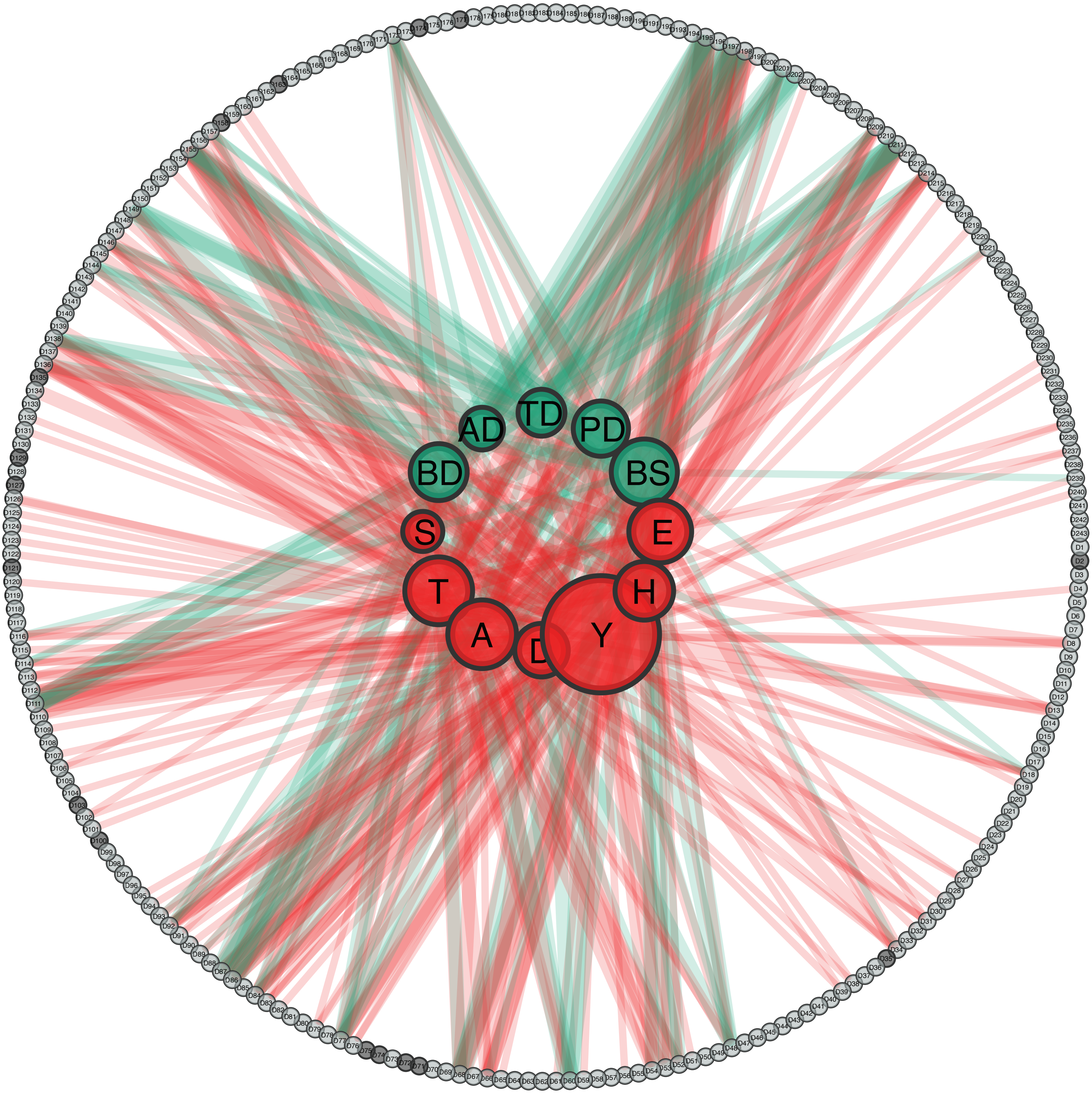

Supplementary figure 11. Network visualisation of phenotypic parameters wild type B, 10 μM

Phenotypic scoring per small molecule concentration illustrated as interaction networks. Inner circles represent phenotypic parameters, with morphology in green and segmentation in red. The size of nodes represents the frequency of scoring >0 for the individual parameter. Interactions to the outer circle represent the scoring of a phenotypic parameter to the associated small molecule treatment (grey circles). A legend of corresponding small molecule names can be found in Supplementary Data 1. Thickness of the interaction lines reflects the magnitude of the score. Dark grey circles represent lethal conditions. The networks are presented with vector graphics, so the details of numbers and interactions can be seen by zooming in.

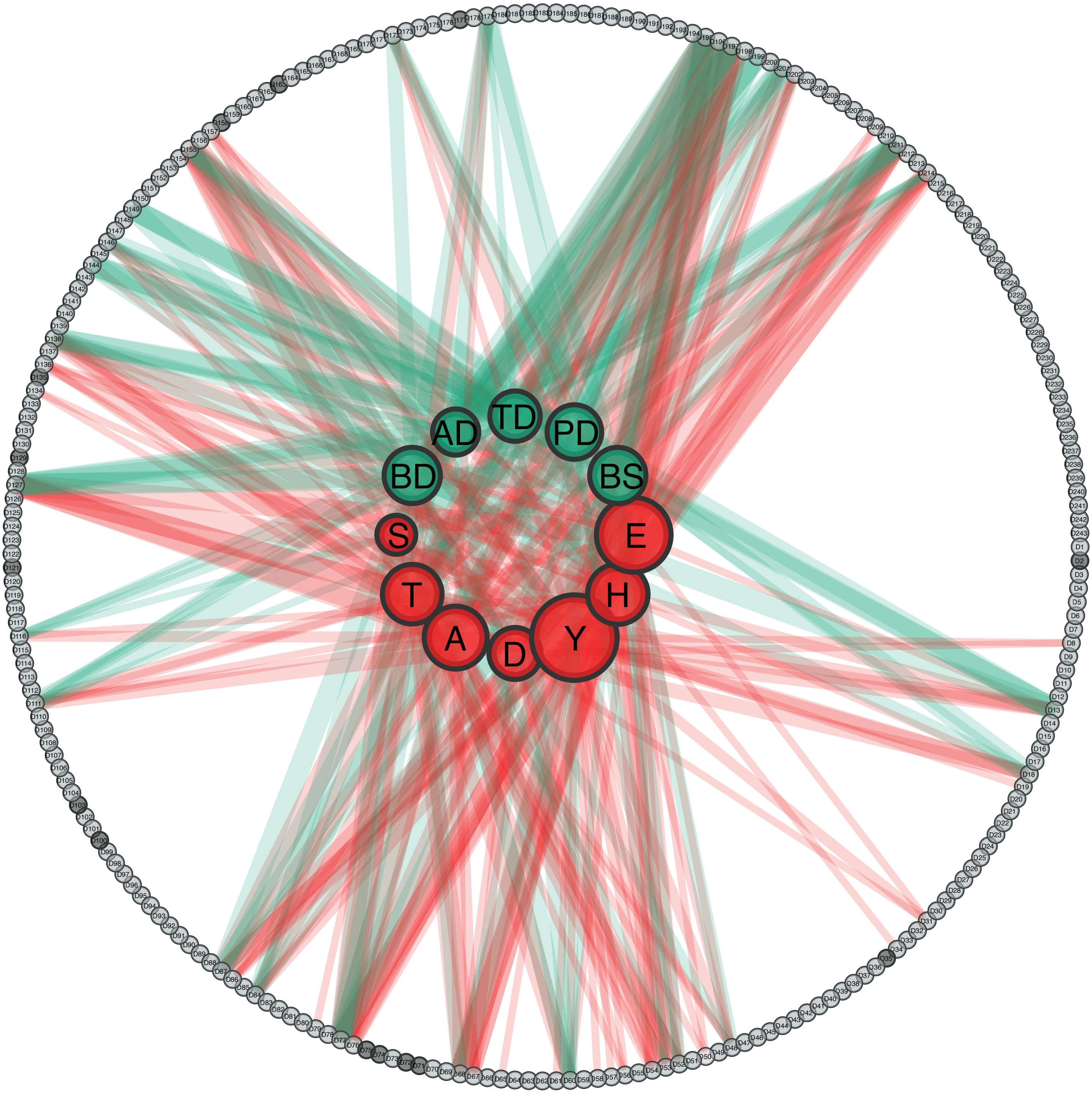

Supplementary figure 12. Network visualisation of phenotypic parameters *her1* mutant, 10 μM

Phenotypic scoring per small molecule concentration illustrated as interaction networks. Inner circles represent phenotypic parameters, with morphology in green and segmentation in red. The size of nodes represents the frequency of scoring >0 for the individual parameter. Interactions to the outer circle represent the scoring of a phenotypic parameter to the associated small molecule treatment (grey circles). A legend of corresponding small molecule names can be found in Supplementary Data 1. Thickness of the interaction lines reflects the magnitude of the score. Dark grey circles represent lethal conditions. The networks are presented with vector graphics, so the details of numbers and interactions can be seen by zooming in.

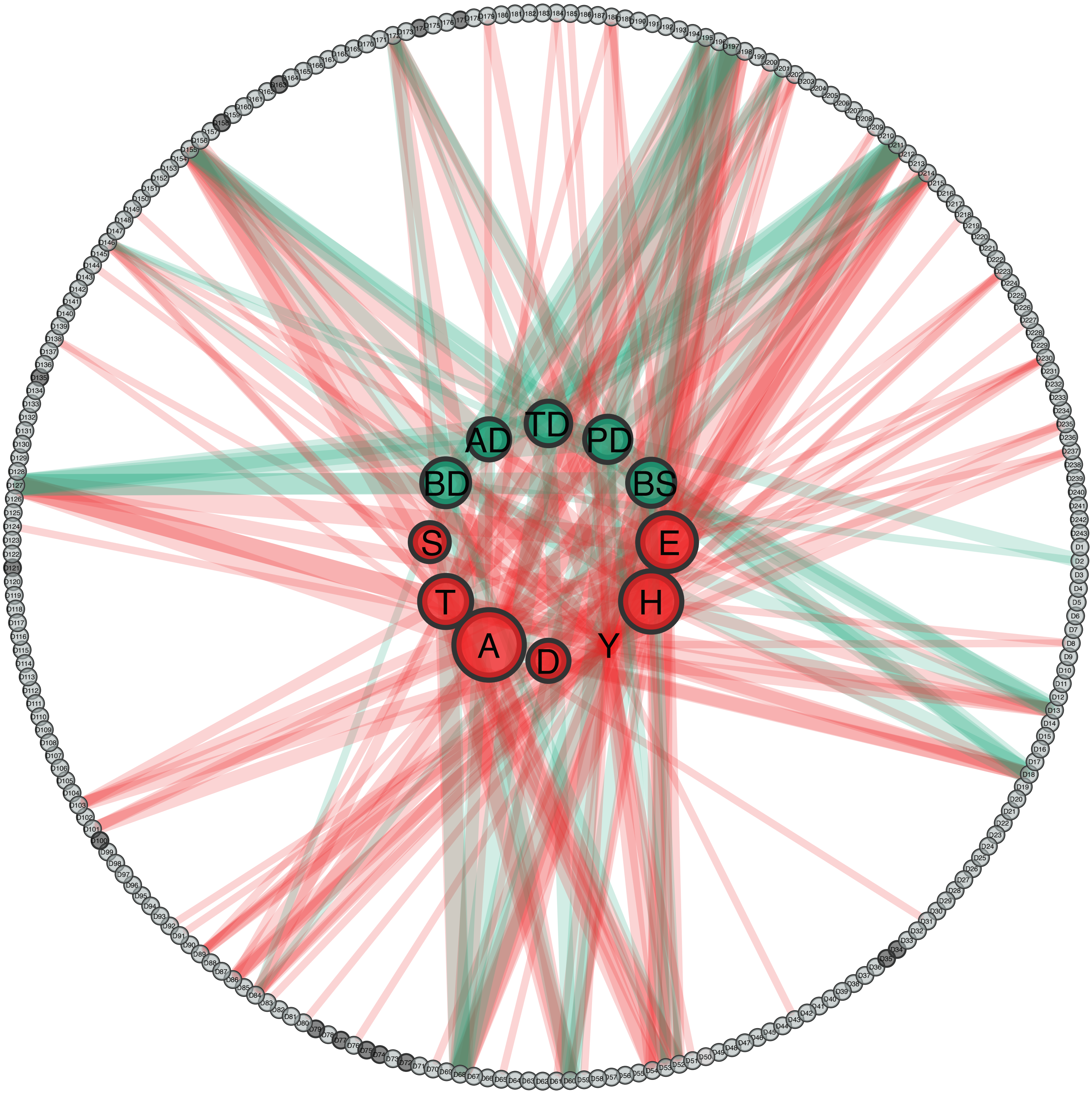

Supplementary figure 13. Network visualisation of phenotypic parameters *hes6* mutant, 10μM

Phenotypic scoring per small molecule concentration illustrated as interaction networks. Inner circles represent phenotypic parameters, with morphology in green and segmentation in red. The size of nodes represents the frequency of scoring >0 for the individual parameter. Interactions to the outer circle represent the scoring of a phenotypic parameter to the associated small molecule treatment (grey circles). A legend of corresponding small molecule names can be found in Supplementary Data 1. Thickness of the interaction lines reflects the magnitude of the score. Dark grey circles represent lethal conditions. The networks are presented with vector graphics, so the details of numbers and interactions can be seen by zooming in.

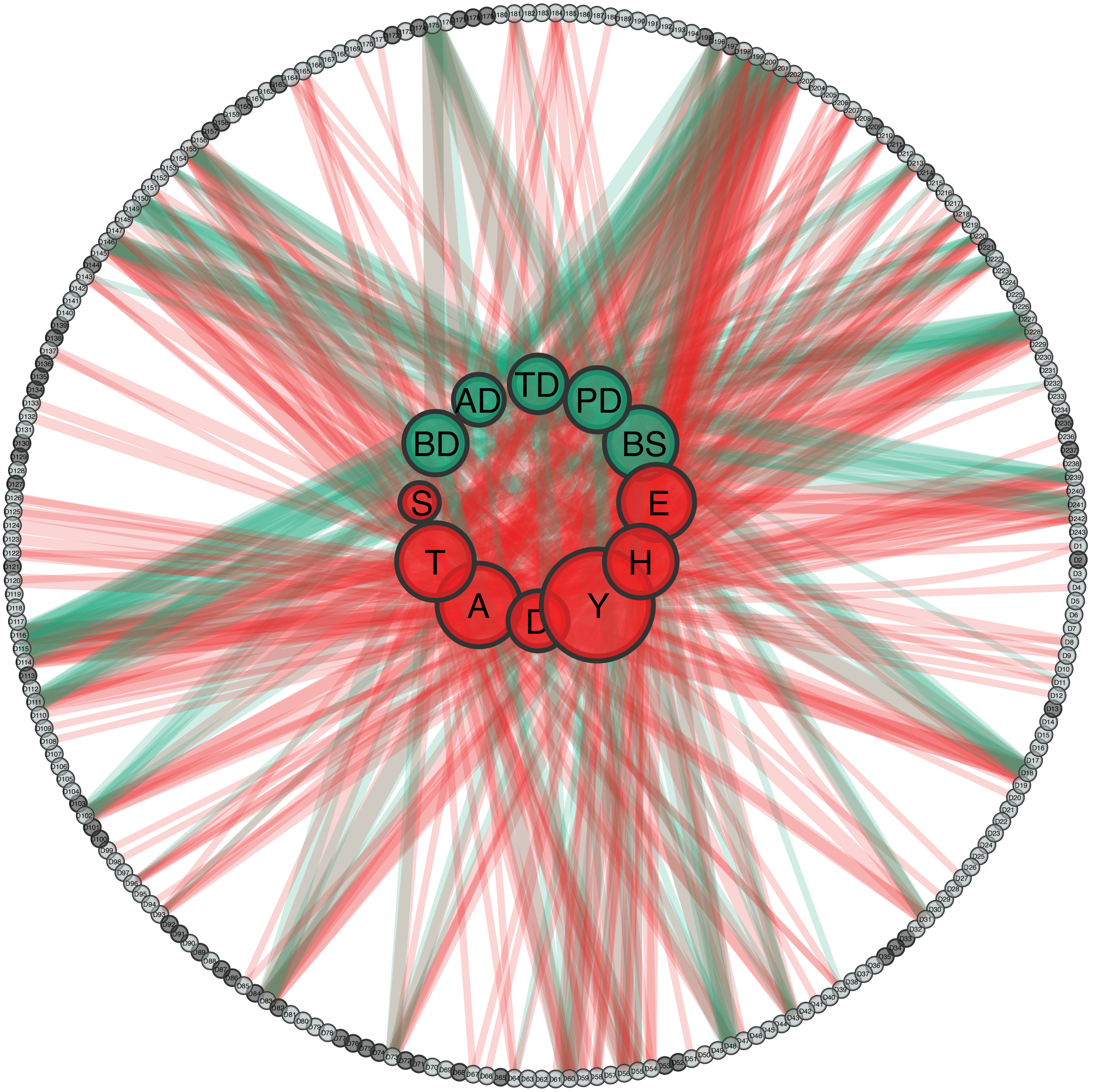

Supplementary figure 14. Network visualisation of phenotypic parameters wild type A, 50 μM

Phenotypic scoring per small molecule concentration illustrated as interaction networks. Inner circles represent phenotypic parameters, with morphology in green and segmentation in red. The size of nodes represents the frequency of scoring >0 for the individual parameter. Interactions to the outer circle represent the scoring of a phenotypic parameter to the associated small molecule treatment (grey circles). A legend of corresponding small molecule names can be found in Supplementary Data 1. Thickness of the interaction lines reflects the magnitude of the score. Dark grey circles represent lethal conditions. The networks are presented with vector graphics, so the details of numbers and interactions can be seen by zooming in.

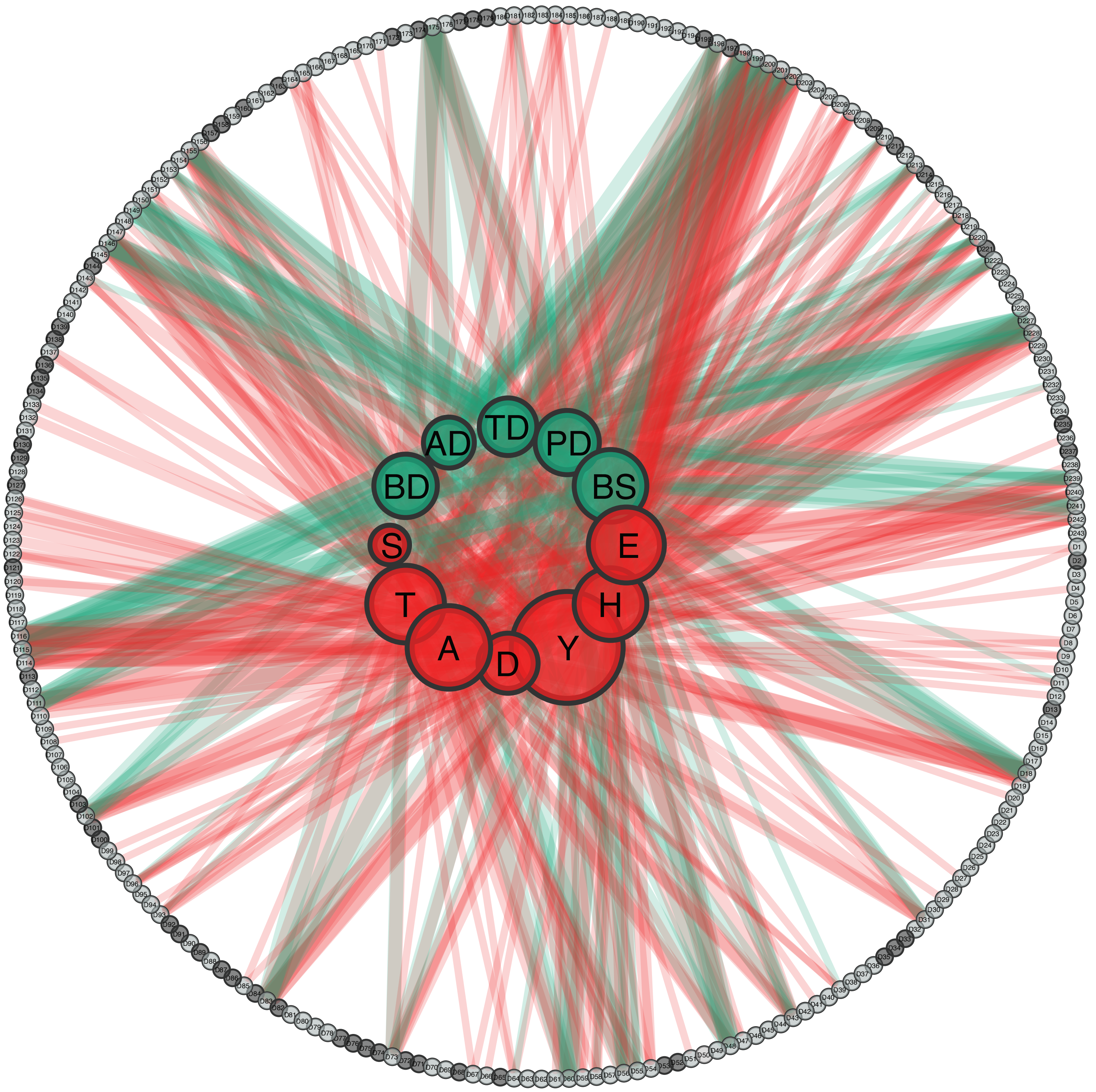

Supplementary figure 15. Network visualisation of phenotypic parameters wild type B, 50 μM

Phenotypic scoring per small molecule concentration illustrated as interaction networks. Inner circles represent phenotypic parameters, with morphology in green and segmentation in red. The size of nodes represents the frequency of scoring >0 for the individual parameter. Interactions to the outer circle represent the scoring of a phenotypic parameter to the associated small molecule treatment (grey circles). A legend of corresponding small molecule names can be found in Supplementary Data 1. Thickness of the interaction lines reflects the magnitude of the score. Dark grey circles represent lethal conditions. The networks are presented with vector graphics, so the details of numbers and interactions can be seen by zooming in.

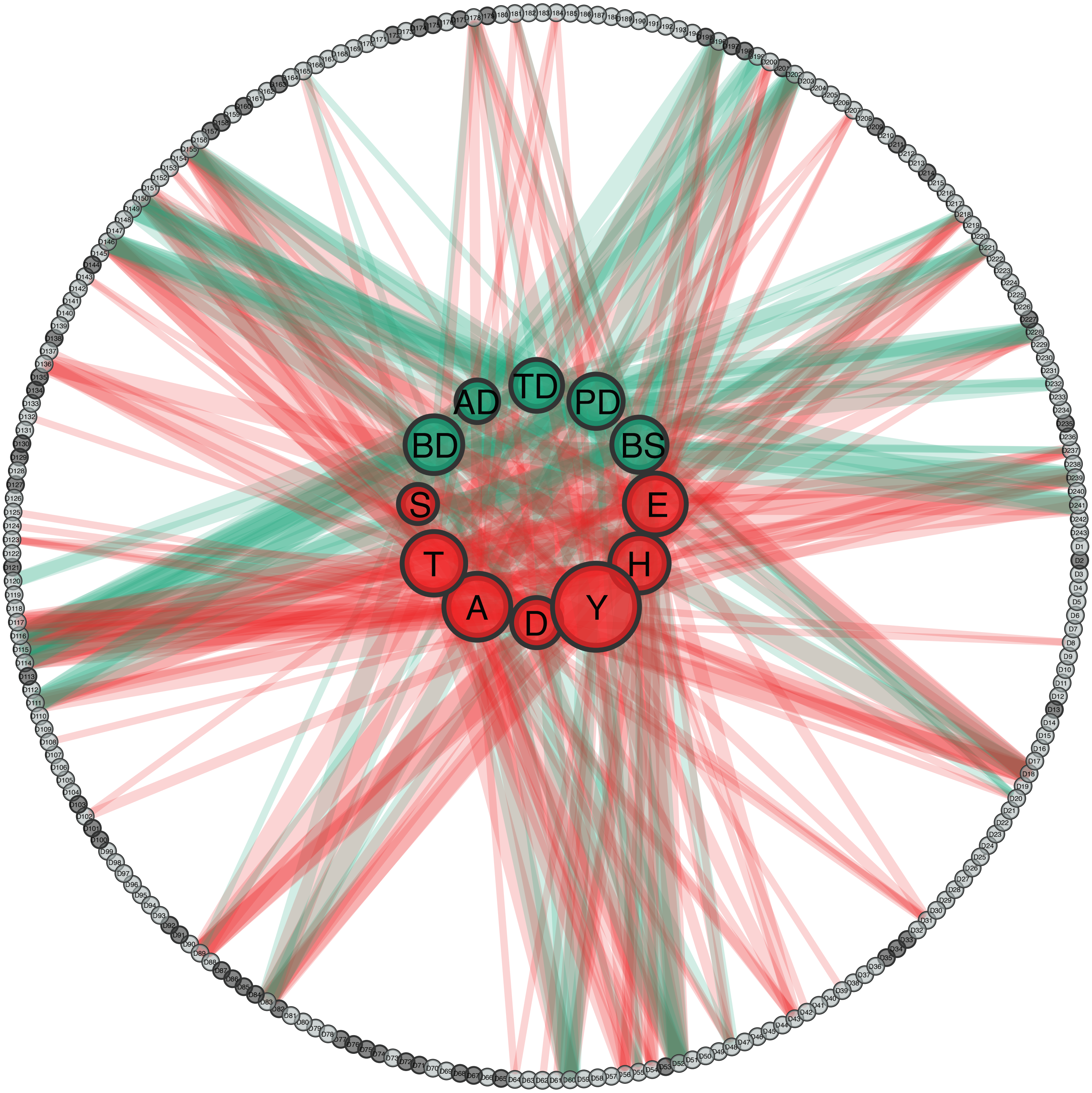

Supplementary figure 16. Network visualisation of phenotypic parameters *her1* mutant, 50 µM

Phenotypic scoring per small molecule concentration illustrated as interaction networks. Inner circles represent phenotypic parameters, with morphology in green and segmentation in red. The size of nodes represents the frequency of scoring >0 for the individual parameter. Interactions to the outer circle represent the scoring of a phenotypic parameter to the associated small molecule treatment (grey circles). A legend of corresponding small molecule names can be found in Supplementary Data 1. Thickness of the interaction lines reflects the magnitude of the score. Dark grey circles represent lethal conditions. The networks are presented with vector graphics, so the details of numbers and interactions can be seen by zooming in.

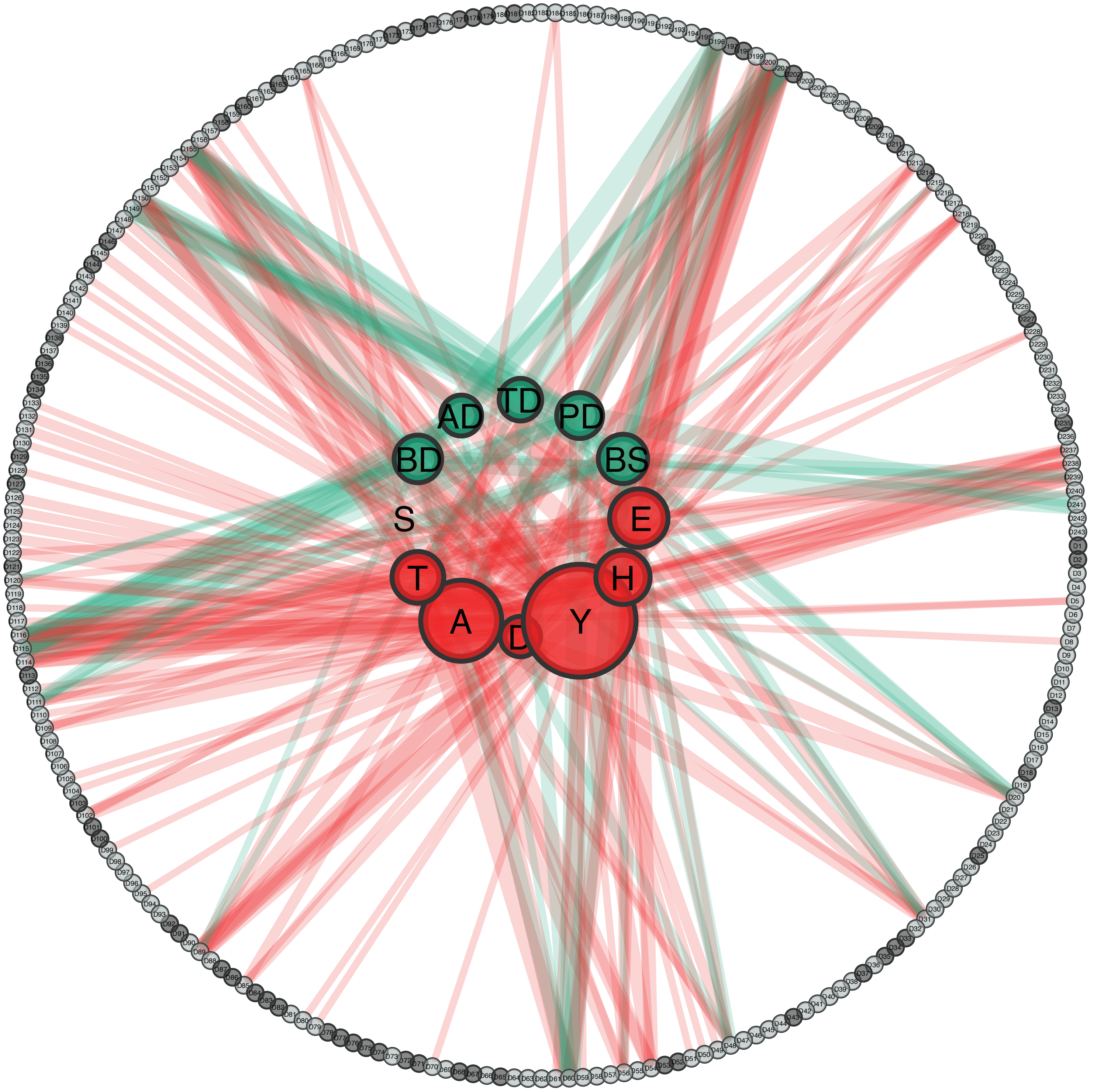

Supplementary figure 17. Network visualisation of phenotypic parameters *hes6* mutant, 50µM

Phenotypic scoring per small molecule concentration illustrated as interaction networks. Inner circles represent phenotypic parameters, with morphology in green and segmentation in red. The size of nodes represents the frequency of scoring >0 for the individual parameter. Interactions to the outer circle represent the scoring of a phenotypic parameter to the associated small molecule treatment (grey circles). A legend of corresponding small molecule names can be found in Supplementary Data 1. Thickness of the interaction lines reflects the magnitude of the score. Dark grey circles represent lethal conditions. The networks are presented with vector graphics, so the details of numbers and interactions can be seen by zooming in.

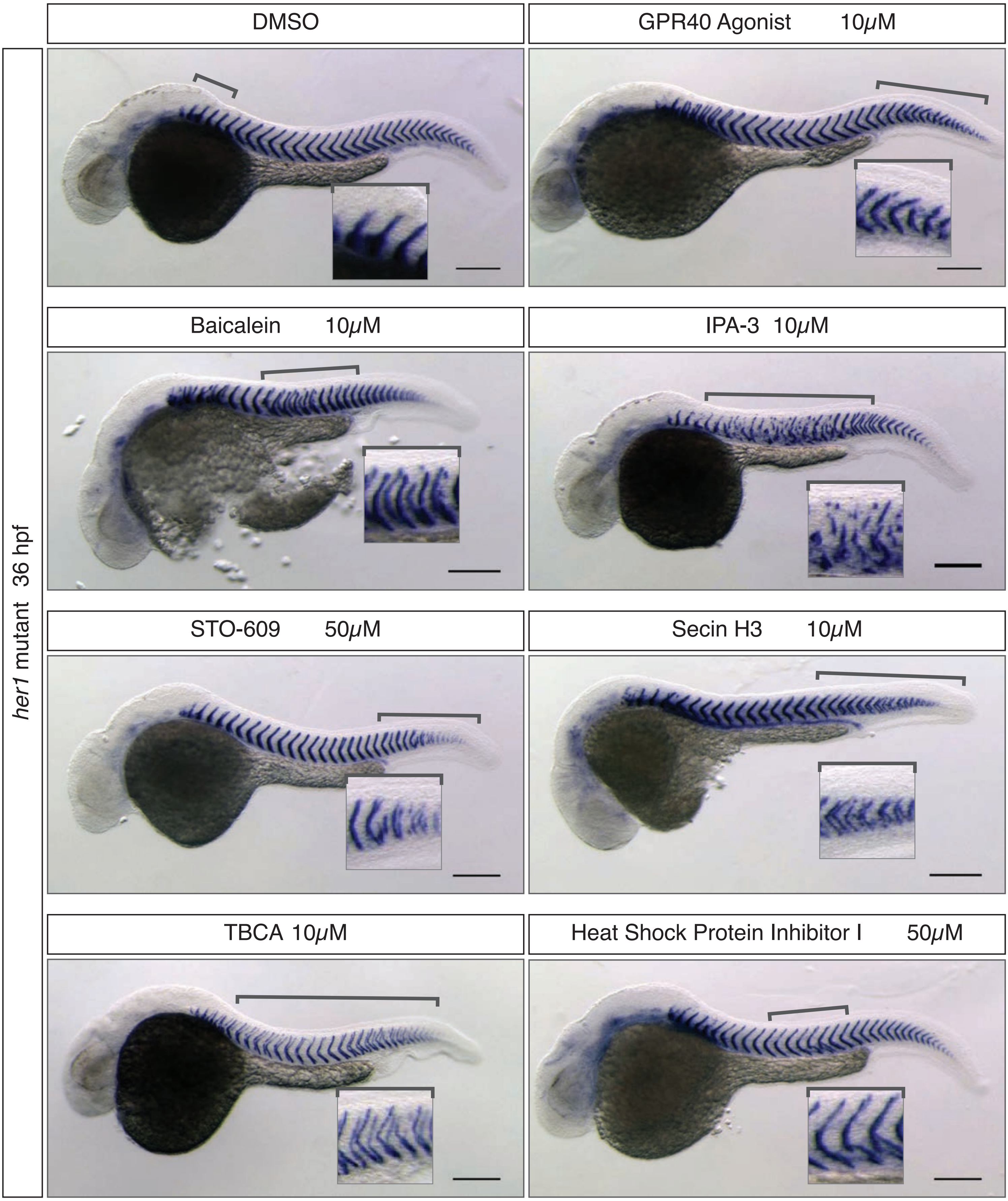

Supplementary figure 18. Direct segmentation phenotypes from *her1* mutants

Identified hit phenotypes with direct segmentation defects according the classification used in Table 1, visible at 36 hpf after *xirp2a in situ* hybridization, in a *her1* mutant background. All treatments are as labelled in the panel above the image. Anterior to the right, posterior to the left. Insets show zoom-in of the segment pattern and defects. Brackets indicate the area of the axis where segment boundary defects occurred.

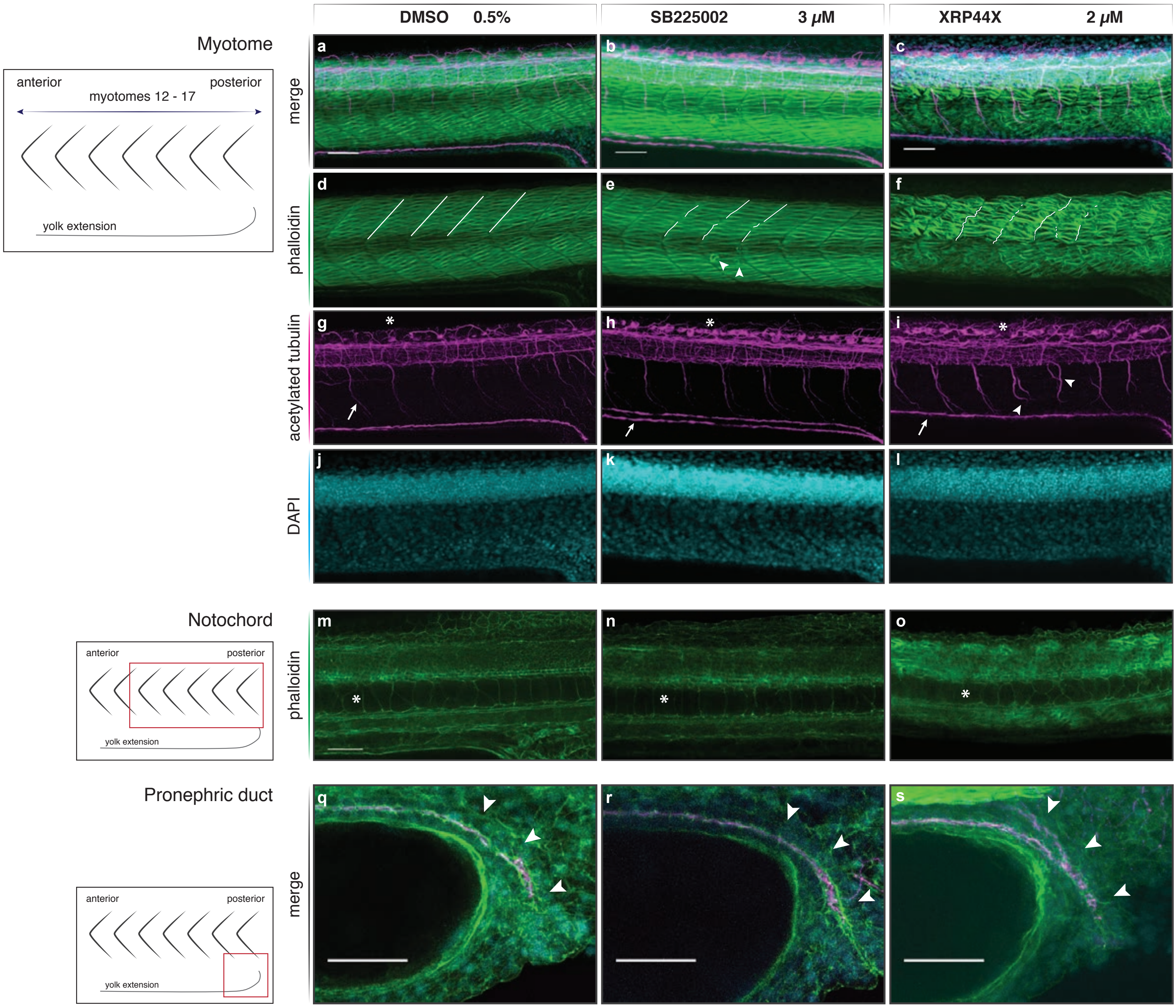

Supplementary figure 19. Trunk myotomes, neurites, notochord and pronephric ducts in embryos treated with SB225002 and XRP44X

Confocal images of DMSO control and small molecule treated embryos at 36 hpf. F-actin visualized with phalloidin (green), acetylated tubulin by immunostaining (magenta) and nuclei with DAPI (cyan). Scale bar is 50 μm. (**a**-**c**) Trunk section through myotomes 12 to 17. Myotomes show F-actin organized in skeletal muscle fibres of the DMSO control (**d**). Muscle fibres are straight and span each myotome from the anterior to the posterior vertical border, highlighted as white lines. (**e**) In 3 μM SB225002 treated embryos, some vertical myotome borders are discontinuous and less straight (white lines). Occasional appearance of round muscle cells and disorganized structures (arrowheads) in myotomes. (**f**) Thicker and shorter muscle fibres in 2 μM XRP44X treated embryos. Muscle fibre structure is disorganized; vertical myotome boundary pattern is disrupted (white lines). (**g**-**i**) Neurons of the peripheral nervous system (asterisks) and pronephric ducts (arrows) developed and extended along the axis in control and small molecule treatments. Ventral axons of primary motor neurons are bent and shorter in XRP44X treated embryos (**i**, arrowheads) compared to DMSO control. (**j**-**l**) Nuclei show a high density at the level of the spinal cord throughout all tested conditions. (**m**-**o**) Cells of the notochord all have vacuoles and are densely packed in control and drug-treated embryos (asterisks). (**q**-**s**) Normal extension of the pronephric ducts until the posterior end of the yolk extension towards the developing anus (arrowheads).

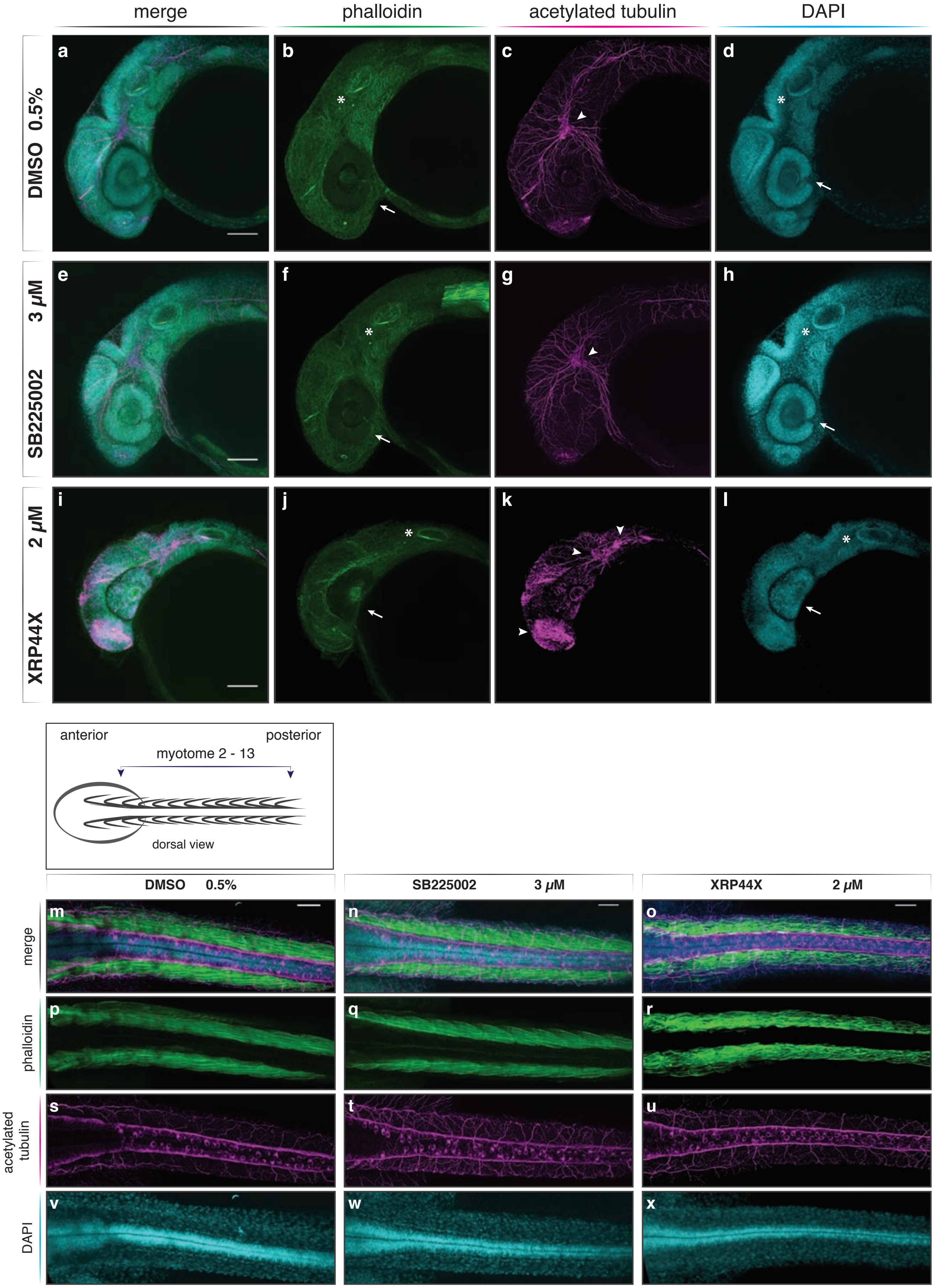

Supplementary figure 20. Head structures, neurites and dorsal view of trunk in SB225002 and XRP44X treated embryos Confocal images of DMSO control and small molecule treated embryos at 36 hpf. F-actin visualized with phalloidin (green), acetylated tubulin by immunostaining (magenta) and nuclei with DAPI (cyan). (**a**-**l**) Maximum projection of a stack of confocal sections of the lateral head of DMSO control and small molecule treated embryos. Scale bar is 100 μm. Control (**a**-**d**) and SB225002 (**e**-**h**) treated embryos show comparable development of main organs and tissue of the head: eyes with lens and retina (arrows), otic vesicles (asterisks) and neurons of the central nervous system (arrowheads). XRP44X treatment (**i**-**l**) led to a reduced eye size and loss of the ventral fissure (**j**), abnormal distribution of acetylated tubulin and accumulation in the head, developing mouth and olfactory organs (asterisks, **k**). (**m**-**x**) Maximum projection of a stack of confocal sections of the dorsal trunk area of DMSO control and small molecule treated embryos. Scale bar is 50 μm. Individual myotome structure is visible in DMSO and SB225002 treated embryos (**p**,**q**), but is less organised in XRP44X treatments (**r**) where individual myotomes are not detectable. (**s**-**u**) Neurons of the peripheral nervous system developed all along the anterior posterior axis in control embryos and all treatments. (**v**-**x**) Nuclei along the axis show a comparable distribution of cells between control and drug treatments.

Supplementary Table 1 Modified SSMD ranking for hit selection

Rank	SSMD	Drug name	Concentration	Genotype
1	1.58113883	gamma-Secretase Inhibitor XXI, Compound E	50	wtA
2	1.46642644	Ras-Net (Elk-3) Pathway Inhibitor, XRP44X	2	Her1 mutant
3	1.392115116	p21-Activated Kinase Inhibitor III, IPA-3	50	Her1 mutant
4	1.290994449	gamma-Secretase Inhibitor XXI, Compound E	50	Her1 mutant
5	1.290994449	gamma-Secretase Inhibitor XXI, Compound E	10	Her1 mutant
6	1.290994449	DAPT	50	wtB
7	1.290994449	DAPT	50	Hes6 mutant
8	1.280150626	DAPT	50	wtA
9	1.245504738	DAPT	50	Her1 mutant
10	1.219875091	DAPT	50	Her1 mutant
11	1.219875091	DAPT	50	Her1 mutant
12	1.219875091	DAPT	50	wtB
13	1.219875091	DAPT	50	wtA
14	1.219875091	TGF-b RI Kinase Inhibitor	50	Her1 mutant
15	1.187339225	DAPT	50	Hes6 mutant
16	1.177621524	p21-Activated Kinase Inhibitor III, IPA-3	10	Her1 mutant
17	1.173691195	PKR Inhibitor	2	Her1 mutant
18	1.146159971	GSK-3 Inhibitor IX	10	wtB
19	1.144344271	SB 225002	10	Her1 mutant
20	1.107018607	DAPT	50	wtA
21	1.100963765	DAPT	50	Hes6 mutant
22	1.088214375	gamma-Secretase Inhibitor XXI, Compound E	10	wtA
23	1.05193031	DAPT	50	wtB
24	1.050525394	gamma-Secretase Inhibitor XXI, Compound E	50	Hes6 mutant
25	1.020620726	gamma-Secretase Inhibitor XXI, Compound E	50	wtB
26	0.99796541	Ras-Net (Elk-3) Pathway Inhibitor, XRP44X	2	Hes6 mutant
27	0.996023841	PP2	10	wtB
28	0.935276002	PP2	2	Hes6 mutant
29	0.87774726	Casein Kinase II Inhibitor III, TBCA	10	Her1 mutant
30	0.84757938	STO-609	50	Her1 mutant
31	0.84757938	GPR30 Agonist, G-1	10	wtA
32	0.845511689	LY-83583	2	Hes6 mutant
33	0.811543068	MEK1,2 Inhibitor II	10	Her1 mutant
34	0.759554525	Syk Inhibitor III	50	wtA
35	0.747904226	Ras-Net (Elk-3) Pathway Inhibitor, XRP44X	2	wtA
36	0.7290038	Wee1 Inhibitor	50	Hes6 mutant
37	0.645497224	Heat Shock Protein Inhibitor I	50	Her1 mutant
38	0.621164027	Wee1 Inhibitor	50	wtB
39	0.617540227	LY-83583	2	wtB
40	0.599329127	JAK3 Inhibitor VI	2	Her1 mutant
41	0.597614305	AG-879	10	Hes6 mutant
42	0.593305566	Wee1 Inhibitor	50	Her1 mutant
43	0.580917864	Ras-Net (Elk-3) Pathway Inhibitor, XRP44X	2	wtB
44	0.564932683	gamma-Secretase Inhibitor XXI, Compound E	10	wtB
45	0.520156487	GPR30 Agonist, G-1	50	wtB
46	0.476731295	GPR30 Agonist, G-1	50	Hes6 mutant
47	0.450225169	MEK1,2 Inhibitor II	10	wtB
48	0.43335139	PP2	50	wtB
49	0.42447636	GPR30 Agonist, G-1	50	Her1 mutant
50	0.40824829	SB 225002	10	wtA
51	0.391230398	GSK-3 Inhibitor IX	50	Her1 mutant
52	0.359721539	PP2	10	Her1 mutant
53	0.358057437	GPR40 Agonist	10	Her1 mutant
54	0.352297424	JAK3 Inhibitor VI	2	wtB
55	0.351104204	SB 225002	10	wtB
56	0.350643437	PP2	10	Hes6 mutant
57	0.350643437	Aristolochic acid	50	Her1 mutant
58	0.334472938	Bafilomycin A1	10	Her1 mutant
59	0.329690237	MEK1,2 Inhibitor II	10	Hes6 mutant
60	0.320530382	PP2	2	Her1 mutant
61	0.315723013	SecinH3	10	Her1 mutant
62	0.314436093	SC-68376	50	Her1 mutant
63	0.314140431	PP2	50	Her1 mutant
64	0.307031719	MEK Inhibitor I	50	wtA
65	0.30429031	17-Allylamino-geldanamycin	10	wtA
66	0.297044263	FGF Receptor Tyrosine Kinase Inhibitor	50	wtA
67	0.294457471	Wee1 Inhibitor	50	wtA
68	0.288675135	STO-609	50	wtB
69	0.286958518	Lck Inhibitor	10	Her1 mutant
70	0.25819889	GSK-3 Inhibitor IX	10	wtA
71	0.25819889	PP2	50	wtA
72	0.256917498	PKR Inhibitor	2	wtB
73	0.253184842	PP2	2	wtA
74	0.243975018	1-Azakenpaullone	10	Her1 mutant
75	0.221403721	SIRT1,2 Inhibitor VIII, Salermide	50	wtB
76	0.203559485	MEK1,2 Inhibitor II	2	wtB
77	0.200401204	Lck Inhibitor	50	Her1 mutant
78	0.176409557	JAK3 Inhibitor VI	10	Hes6 mutant
79	0.157851017	SC-68376	50	wtA
80	0.149181744	Lck Inhibitor	50	wtA
81	0.148087219	Cyclopamine	10	wtB
82	0.142566487	Roscovitine	50	wtB
83	0.140028008	FGF Receptor Tyrosine Kinase Inhibitor	50	Hes6 mutant
84	0.130410133	FGF Receptor Tyrosine Kinase Inhibitor	10	Her1 mutant
85	0.121446542	Cyclopamine	50	wtB
86	0.113227703	GPR40 Agonist	50	Her1 mutant
87	0.104031297	17-Allylamino-geldanamycin	10	Hes6 mutant
88	0.090834052	Lck Inhibitor	10	wtB
89	0.087437177	SU-5402	50	wtA
90	0.085875673	FGF Receptor Tyrosine Kinase Inhibitor	50	Her1 mutant
91	0.081649658	1-Azakenpaullone	10	wtB
92	0.070220841	MEK Inhibitor I	50	wtB
93	0.070220841	TNF-alpha Antagonist III, R-7050	50	wtB
94	0.063144603	FGF Receptor Tyrosine Kinase Inhibitor	2	wtB
95	0.056943324	Bafilomycin A1	10	Hes6 mutant
96	0.054849098	MEK1,2 Inhibitor II	10	wtA
97	0.051598516	VEGF Receptor Tyrosine Kinase Inhibitor II	50	wtA
98	0.044919442	SU-5402	10	Her1 mutant
99	0	Aristolochic acid	50	Hes6 mutant
100	0	Baicalein	2	wtB
101	0	Bay 11-7082	2	wtB
102	0	Cyclopamine	2	wtB
103	0	PKR Inhibitor	2	wtA
104	0	Lck Inhibitor	10	wtA
105	0	JAK3 Inhibitor VI	2	wtA
106	0	FGF Receptor Tyrosine Kinase Inhibitor	10	wtA
107	0	Manumycin A	10	wtA
108	0	SP-600125	2	wtA
109	-0.056943324	Z-Guggulsterone	50	Her1 mutant
110	-0.0747853	Baicalein	10	Her1 mutant
111	-0.099014754	Diacylglycerol Kinase Inhibitor II	50	Her1 mutant
112	-0.102062073	A77 1726	10	wtB
113	-0.114108866	Lck Inhibitor	50	Hes6 mutant
114	-0.119865825	IKK Inhibitor X	50	wtA
115	-0.121987509	p38 MAP Kinase Inhibitor	50	Her1 mutant
116	-0.142566487	SC-68376	50	wtB
117	-0.162221421	17-Allylamino-geldanamycin	10	Her1 mutant
118	-0.169515876	PP2	10	wtA
119	-0.18590915	TNF-alpha Antagonist III, R-7050	50	Hes6 mutant
120	-0.208062595	A77 1726	10	Her1 mutant

Supplementary Table 2 Mean difference of segmentation and morphology parameters for hit selection

Drug name	2 µM wt_A	2 µM wt_B	2 µM *her1* mutant	2 µM *hes6* mutant	10 µM wt_A	10 µM wt_B	10 µM *her1* mutant	10 µM *hes6* mutant	50 µM wt_A	50 µM wt_B	50 µM *her1* mutant	50 µM *hes6* mutant
1-Azakenpaullone		0.083	0.083			0.083	0.333					
17-Allylamino-geldanamycin					0.500			0.167				
Aristolochic acid											0.250	
Bafilomycin A1							0.583	0.083				
Casein Kinase II Inhibitor III, TBCA							1.167					
Cyclopamine						0.167				0.167		
FGF Receptor Tyrosine Kinase Inhibitor		0.083				0.333	0.167		0.500	0.333	0.167	0.167
GPR30 Agonist, G-1					0.833					0.833	0.667	0.750
GPR40 Agonist							0.333				0.167	
GSK-3 Inhibitor IX					0.167	1.583					0.500	
Heat Shock Protein Inhibitor I											0.667	
JAK3 Inhibitor VI		0.583	0.833					0.250				
LY-83583		0.750		1.083								
Lck Inhibitor						0.083	0.417		0.250		0.333	
MEK Inhibitor I									0.417	0.083		
MEK1/2 Inhibitor II		0.250			0.083	0.500	1.167	0.250				
PKR Inhibitor		0.333	1.500			0.333						
PP2	0.167	0.083	0.250	1.083		1.250	0.417	0.500	0.333	0.667	0.500	
Ras-Net (Elk-3) Pathway Inhibitor, XRP44X	1.167	0.583	1.917	1.167								
SB 225002					0.667	0.417	1.833					
SC-68376					0.167				0.250		0.583	
STO-609									0.167	0.167	0.833	
SecinH3							0.417					
TGF-b RI Kinase Inhibitor					0.167						1.667	
Wee1 Inhibitor									0.500	1.083	1.167	1.083
p21-Activated Kinase Inhibitor III, IPA-3							1.750				1.667	
γ-Secretase Inhibitor XXI, Compound E					1.500	1.000	2.000		2.000	1.667	2.000	1.750
AG-879								0.500				
DAPT_1									1.667	1.500	1.667	1.333
DAPT_2									1.667	1.667	1.667	1.250
DAPT_3									1.917	2.000	1.833	0.667
Roscovitine										0.167		
SIRT1/2 Inhibitor VIII, Salermide										0.167		
SU5402_2							0.083		0.167			
Syk Inhibitor III									0.500			
TNF-α Antagonist III, R-7050										0.083		
VEGF Receptor Tyrosine Kinase Inhibitor II									0.083			
Apoptosis Inhibitor II, NS3694				0.167								
E3 medium_21		0.083										
E3 medium_29						0.083						

Supplementary Table 3 Genotyping primer sequences

	Primer name	Primer sequence (5' to 3')	T_m
her1	zf-her1-236-outer-for	TACGTCATTGACACCTCGTC	57°C
	zf-her1-238-inner-for	CCATATAGTATTCCAGGTTGTGTC	57°C
	zf-her1-239-inner-rev	TGTAAAACGACGGCCAGTGGTTTGCAAGGGACTTTAAC	57°C
	zf-her1-237-outer-rev	AGGAAACAGCTATGACCATGCTTACTTGTATAAGCTACAATTGAC	57°C
hes6	hes6-701-for	AGCAACACTCACGACGAGGATTA	55°C
	hes6-1522-rev	CAGAACAAATGTCGTCGCTGGAG	55°C
	5'LTR-600	AAGTCGGATGCAACTGCAAGAAGG	57°C
