# Supplementary material for: Small molecule screen in embryonic zebrafish using modular variations to target segmentation
Source: Nat Commun. 2017 Dec 1;8:1901. doi: 10.1038/s41467-017-01469-5 (PMC5711842; doi:10.1038/s41467-017-01469-5)
Supplement: Supplementary file 3 — Description of Additional Supplementary Files [file 41467_2017_1469_MOESM3_ESM.pdf]

Description of Additional Supplementary Files

File Name: Supplementary Data 1
Description: XL file of the compounds used in the screen
